# Global Transcriptional Analyses of the Wnt-Induced Development of Neural Stem Cells from Human Pluripotent Stem Cells

**DOI:** 10.3390/ijms22147473

**Published:** 2021-07-12

**Authors:** Bing-Chun Liu, Fang-Yuan Liu, Xin-Yue Gao, Yang-Lin Chen, Qiao-Qiao Meng, Yong-Li Song, Xi-He Li, Si-Qin Bao

**Affiliations:** 1 The State Key Laboratory of Reproductive Regulation and Breeding of Grassland Livestock, College of Life Sciences, Inner Mongolia University, Hohhot 010020, China; liubingchun@mail.imu.edu.cn (B.-C.L.); liufy@mail.imu.edu.cn (F.-Y.L.); gaoxinyue@mail.imu.edu.cn (X.-Y.G.); chenyanglincyl@mail.imu.edu.cn (Y.-L.C.); mengqiao@mail.imu.edu.cn (Q.-Q.M.); ylsong@imu.edu.cn (Y.-L.S.); lixh@imu.edu.cn (X.-H.L.); 2 Research Center for Animal Genetic Resources of Mongolia Plateau, College of Life Sciences, Inner Mongolia University, Hohhot 010020, China; 3 Inner Mongolia Saikexing Institute of Breeding and Reproductive Biotechnology in Domestic Animal, Hohhot 011517, China

**Keywords:** human pluripotent stem cells, neural stem cells, Wnt signaling, spinal cord

## Abstract

The differentiation of human pluripotent stem cells (hPSCs) to neural stem cells (NSCs) is the key initial event in neurogenesis and is thought to be dependent on the family of Wnt growth factors, their receptors and signaling proteins. The delineation of the transcriptional pathways that mediate Wnt-induced hPSCs to NSCs differentiation is vital for understanding the global genomic mechanisms of the development of NSCs and, potentially, the creation of new protocols in regenerative medicine. To understand the genomic mechanism of Wnt signaling during NSCs development, we treated hPSCs with Wnt activator (CHIR-99021) and leukemia inhibitory factor (LIF) in a chemically defined medium (N2B27) to induce NSCs, referred to as CLNSCs. The CLNSCs were subcultured for more than 40 passages in vitro; were positive for AP staining; expressed neural progenitor markers such as *NESTIN*, *PAX6*, *SOX2*, and *SOX1*; and were able to differentiate into three neural lineage cells: neurons, astrocytes, and oligodendrocytes in vitro. Our transcriptome analyses revealed that the Wnt and Hedgehog signaling pathways regulate hPSCs cell fate decisions for neural lineages and maintain the self-renewal of CLNSCs. One interesting network could be the deregulation of the Wnt/β-catenin signaling pathway in CLNSCs via the downregulation of c-MYC, which may promote exit from pluripotency and neural differentiation. The Wnt-induced spinal markers *HOXA1-4*, *HOXA7*, *HOXB1-4,* and *HOXC4* were increased, however, the brain markers *FOXG1* and *OTX2*, were absent in the CLNSCs, indicating that CLNSCs have partial spinal cord properties. Finally, a CLNSC simple culture condition, when applied to hPSCs, supports the generation of NSCs, and provides a new and efficient cell model with which to untangle the mechanisms during neurogenesis.

## 1. Introduction

Over the past several years, neural stem cell transplantation has been widely used in neurological diseases, such as stroke [1,2], amyotrophic lateral sclerosis [3], spinal cord injury [4,5], and neural stem cell based anticancer gene therapy [6]. There are various resources for manufacturing NSCs in vitro, such as NSCs differentiated from embryonic stem cells (ESCs) [7,8,9] or induced pluripotent stem cells (iPSCs) [10,11,12,13,14], as well as NSCs reprogramed from somatic stem cells [15,16,17,18], and these NSCs could potentially be used for neural stem cell transplantation therapies.

Growth factors such as epidermal growth factor (EGF) and fibroblast growth factor 2 (FGF2) can stimulate neurogenesis [19,20] and derive neural progenitors and neural stem cells from human embryonic stem cells (hESCs) [21,22,23,24]. Another classic strategy is to obtain neural stem cells through a dual-inhibitor approach, which is treatment with Noggin or LDN-193189 and SB-431542 (inhibitors of the SMAD signaling pathway, Noggin and LDN-193189 are inhibitors of bone morphogenetic proteins, while SB-431542 is an inhibitor of the transforming growth factor-β type I receptor/ALK5) [25,26]. Dual SMAD inhibition blocks the BMP and TGFβ signaling pathways and, when transduced by SMADs, results in neural differentiation [26]. The iPSCs were first proposed in 2006 by Takahashi and Yamanaka through the overexpression of four core transcription factors in mouse somatic cells [27] and human induced pluripotency stem cells (hiPSCs) that were further derived from somatic cells in 2007 [28,29]. Since then, numerous studies have reported NSCs induced from iPSCs that utilize comparable protocols to hESCs [30,31]. Methods based on those described above have been studied and optimized to improve the differentiation efficiency—in turn, brain region-specific and spinal cord region-specific neural progenitor/stem cells have been obtained, respectively [32,33,34,35]. Fetal brain region-specific NSCs highly express *SIX3*, *FOXG1*, *EN2*, *PAX5*, and *GSX2* under LDN-193189, SB-431542, EGF, and FGF conditions, while spinal cord NSCs express the homeobox (*HOX*) genes by the activation of Wnt and FGF2/8 signaling in conjunction with the dual inhibition of SMAD signaling [34]. Wnt signaling controls both stem cell maintenance and cell fate decisions. The maintenance of pluripotency in embryonic stem cells through the activation of Wnt signaling was originally proposed in 2004 [36] and has been validated by other studies [37,38]. On the other hand, studies have indicated that Wnt signaling results in the varied differentiation tendency of hPSCs [39]. A recent study showed that CHIR-99021 played a critical role in the formation of gastruloids [40]. The critical role Wnt signaling plays through different receptors in NSCs has been previously reported to have multiple functions. For example, Wnt/β-catenin canonical signaling activates *PAX6* expression through the β-catenin/LEF1 fusion protein and boosts neurogenesis in the development of the mouse brain [41], and the degradation of β-catenin causes the inactivation of Wnt/β-catenin canonical signaling to maintain NSCs proliferation [42]. Nevertheless, the regulators and regulatory mechanisms of the Wnt pathway in NSCs remain debatable. A recent in vivo study suggested that the region-specific NSC phenotype is more conducive to the treatment of this disease or injury area, especially for the precise repair of similar damaged cell types [43]. However, the current methods to acquire region-specific NSCs usually go through a series of different stages of cocktail induction, which is time consuming and cost effective, and the combination of a multi-factor cocktail culture system poses difficulties for determining the mechanism of neural differentiation and maintenance, which is unfavorable to the further research of the events that occur during neurogenesis. Therefore, a more simple and rapid method to gain region-specific NSCs still needs to be developed to meet the increasing demand for clinical stem cell therapy and provide a good model to investigate the mechanisms underlying the differentiation of hPSCs into NSCs.

Here, we induced NSCs from hPSCs (hESCs and hiPSCs) in chemically defined conditions by a combination of CHIR-99021 and LIF. The CLNSCs generated by our method tended to express the neural markers *SOX2*, *PAX6*, *NESTIN*, *SOX1*, *MSI1*, and also have spinal cord regional expression patterns, as indicated by the positive expression of *HOXA1-4*, *HOXA7*, *HOXB1-4*, and *HOXC4* and the negative expression of forebrain marker *FOXG1*. Interestingly, the maintenance of self-renewing CLNSCs with a spinal cord expression pattern is likely due to non-canonical Wnt signaling and not canonical Wnt/β-catenin signaling. The strategy used to derive defined NSCs in an efficient, simple, and reproducible manner from hPSCs provides a powerful model of neurological disorders in vitro and may support further applications in neural stem cell therapy and regenerative medicine.

## 2. Results

### 2.1. Combination of CHIR-99021 and LIF Can Generate NSCs from hPSCs

**Figure 1:**
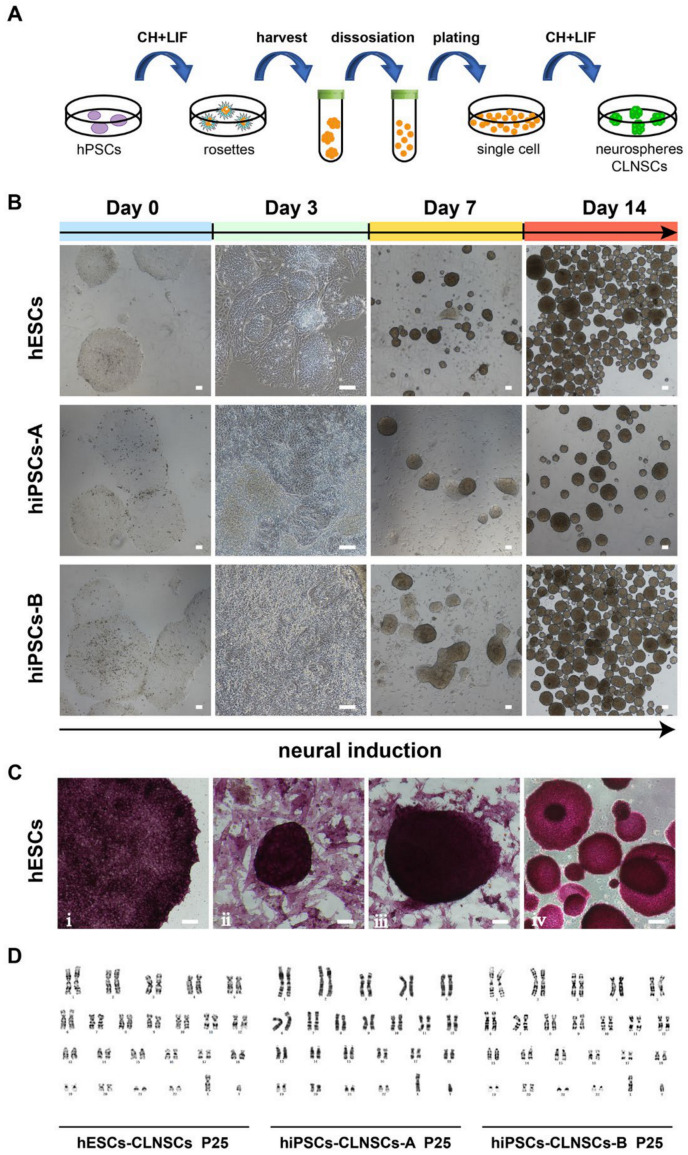
Generation of CLNSCs derived from hPSCs. (**A**) Schematic diagram of hPSCs to CLNSCs differentiation. (**B**) Morphology changes in three hPSC cell lines during neural induction at different times, scale bar: 100 μm. (**C**) AP staining of hESCs cultured in CL medium. ⅰ: Day 0; ⅱ: Day 3; ⅲ: Day 7; ⅳ: Day 14. Scale bar: 100 μm. (**D**) Karyotype analysis of the CLNSCs derived from hPSCs (Passage 25), showing normal karyotype.

The hPSCs, including hESCs and iPSCs, were cultured in mTeSR medium under feeder-free conditions then replaced to N2B27 media supplemented with CHIR-99021 and LIF for the derivation of NSCs; we named these induced neural stem cells CLNSCs. The whole culture procedure is shown by a schematic diagram (Figure 1A). After 2~3 days of culture, a neural rosette structure was observed in the neural induction medium, as described above (named CL medium). The neural precursor marker NESTIN and neuroepithelial marker N-cadherin (Figure S1A) were shown in a neural rosette structure, much like those reported previously [44,45,46]. The neurospheres of induced cells were found in culture at 5–7 days, collected by centrifuge, and dissociated using Accutase to single cells for passage. The induced cells began to grow adherently on the plate, adherent clones appeared within 2 or 3 days, and suspension neurospheres formed after 5 to 6 days.

There was a series of dramatic changes in the hPSC cell morphology in CL medium, including neural rosettes, monolayers of cells, and neurospheres (Figure 1B); the same trend was present in all three cell lines (hESCs, hiPSCs-A, hiPSCs-B). Positive AP staining at different stages of neural induction were shown (Figure 1C), indicating that CLNSCs derived from hPSCs remained for stem cell identity. A single cell could grow to a neurosphere in 6–7 days in CL medium. Neurospheres were passaged every 6~7 days by a single-cell digestion procedure and maintained over 40 passages with a stable karyotype (Figure 1D, Figure S1B). Taken together, our findings show that our protocol was more effective for CLNSCs derived from hPSCs.

### 2.2. Characteristic of NSCs Derived from hPSCs

**Figure 2:**
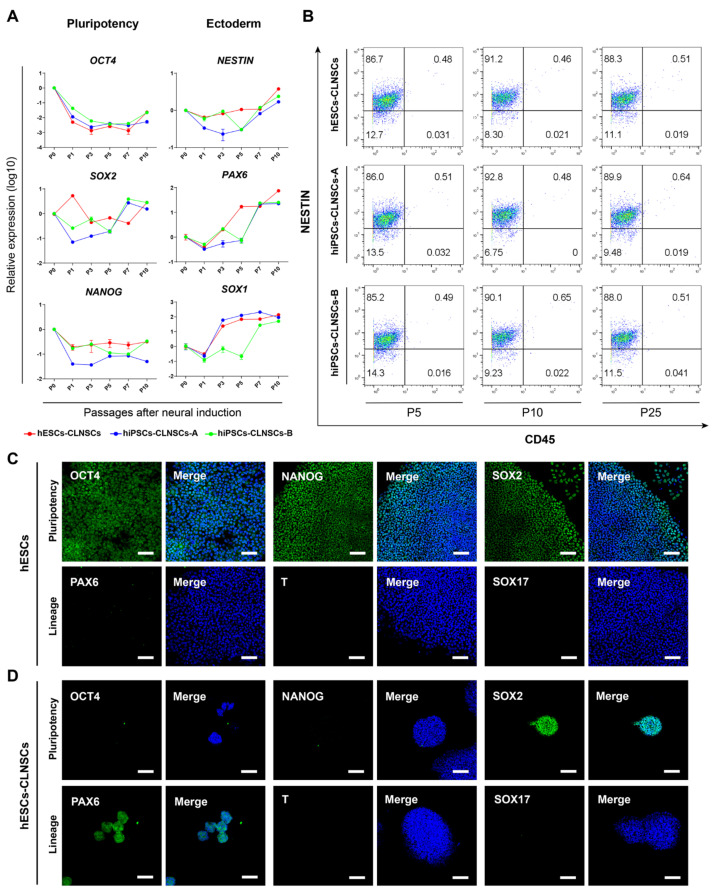
Characteristics of hPSCs-derived NSCs. (**A**) qRT-PCR analysis of *OCT4*, *SOX2*, *NANOG*, *NESTIN*, PAX6, *SOX1* in hPSCs after neural induction. Relative mRNA expression values were normalized to *GAPDH*. Error bar indicates the mean ± SD of three independent experiments in duplicate. (**B**) Detection of neural marker NESTIN and pan-leukocyte marker CD45 expression by flow cytometry in Passage 5, Passage 10, and Passage 25 of CLNSCs derived from hPSCs. (**C**) Immunofluorescence staining for pluripotent markers OCT4, SOX2, and NANOG and lineage markers PAX6, T, and SOX17 in hESCs (Passage 28), scale bar: 100 μm. (**D**) Immunofluorescence staining for pluripotent markers OCT4, NANOG, and SOX2 as well as neural marker PAX6, mesodermal marker T, and endodermal marker SOX17 in hESC-derived CLNSCs (Passage 25), scale bar: 100 μm.

With an increase in the passage number of CLNSCs, we found that the mRNA expression levels of the pluripotent markers *OCT4* and *NANOG* were gradually downregulated while *SOX2* was maintained after some fluctuations in all three hPSC cell lines, indicating an exit from the pluripotent state (Figure 2A). In addition, the mRNA expression of the neural stem cell markers *NESTIN*, *PAX6*, and *SOX1* was dramatically upregulated as the passage number increased (Figure 2A). Flow cytometry demonstrated that a high ratio of neural stem cell marker NESTIN-positive cells remained relatively stable during the different passages (P5, P10, and P25) of CLNSCs (Figure 2B). Immunostaining showed that the hESCs expressed pluripotent markers OCT4, SOX2, and NANOG, but without ectoderm marker PAX6, mesoderm marker T (TBXT or Brachyury), or endoderm marker SOX17 expression (Figure 2C). The positive expression of neural markers PAX6 and SOX2 as well as the negative expression of OCT4 and NANOG were revealed by immunostaining in Passage 25 of the CLNSCs, and these cells also did not express mesoderm and endoderm lineage markers T and SOX17 (Figure 2D). Additionally, the immunostaining of hiPSCs and hiPSCs-CLNSCs is shown in Figure S2A–D. Taken together, we concluded that the CLNSCs derived from hPSCs exhibited NSCs properties. This is consistent with the data concerning the NSCs described before [47,48].

### 2.3. CLNSCs Display Multipotency during Differentiation

**Figure 3:**
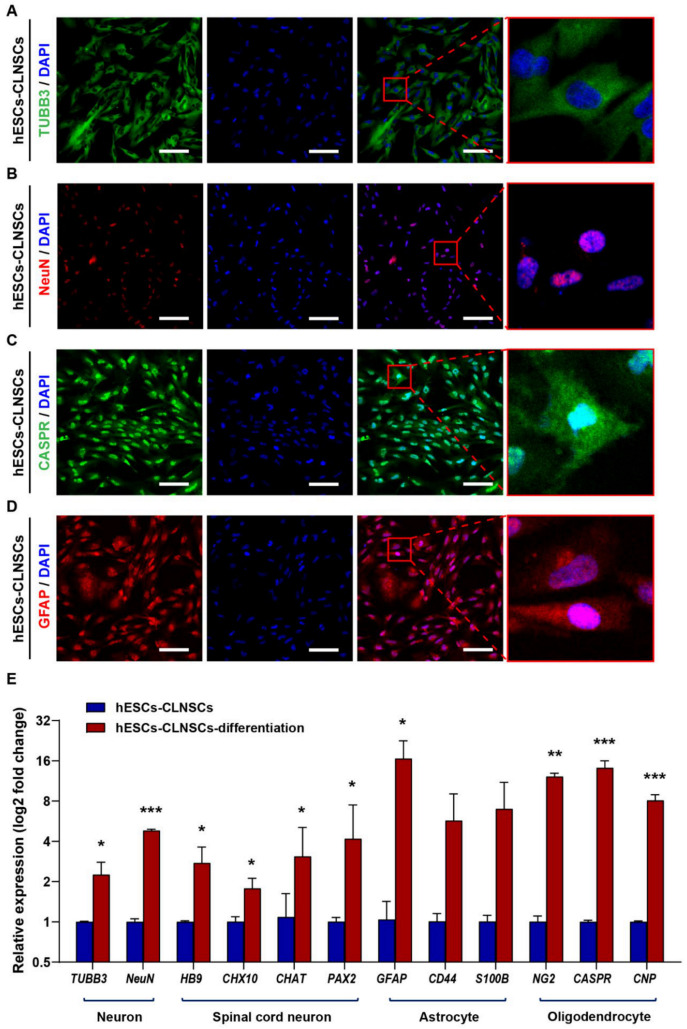
The hESCs-derived CLNSCs differentiated to neurons, astrocytes, and oligodendrocytes. (**A**) Specific immunostaining for neuronal marker TUBB3 (green) was confined into the cytoplasm, scale bar: 100 μm. Right picture shows a magnification of the merge at a ratio of 1:50. (**B**) Specific immunostaining of neuronal nuclei marker NeuN (red), scale bar: 100 μm; right picture shows a magnification of the merge at a ratio of 1:50. (**C**) Immunostaining for oligodendrocyte marker CASPR (green), scale bar: 100 μm. Right picture shows a magnification of the merge at a ratio of 1:50. (**D**) Immunostaining for astrocytic marker GFAP (red), scale bar: 100 μm. Right picture shows a magnification of the merge at a ratio of 1:50. (**E**) qRT-PCR analysis of neural gene expression in hESCs-CLNSCs and their differentiated cells (Passage 28) after 12 days of induction of differentiation. Data represent mean values of 3 independent experiments. Error bars indicate SD, significant differences were determined using Student’s *t*-test; * *p* < 0.05; ** *p* < 0.01; *** *p* < 0.001 for indicated comparisons.

The multipotent potential of CLNSCs was assessed by their capacity to generate the three distinct neural lineages: neurons, astrocytes, and oligodendrocytes. The CLNSCs were observed after 8 days of culture (Figure 3A–D) using the NeuroCult™ NS-A Differentiation Kit (StemCell Technologies). The neurons were labeled with TUBB3 (βIII-tubulin), NeuN and the astrocytes were labeled by GFAP, and oligodendrocytes were labeled by CASPR. The CLNSCs derived from both hESCs and iPSCs showed a multipotent ability to differentiate into the three neural lineages. The results of immunostaining for the neural lineage markers of CLNSCs derived from iPSCs-A and iPSCs-B were similar to those of the CLNSCs derived from hESCs (Figure S3A,B). To further analyze CLNSC differentiation, qRT-PCR was used to measure the mRNA expression of neural markers, including neuron markers *TUBB3* and *NeuN*, spinal cord motoneuron markers *HB9* and *CHAT*, excitatory interneuron marker *CHX10*, and inhibitory interneuron *PAX2.* CLNSC differentiated cells after 12 days of culture expressed higher levels of neural lineage markers compared with CLNSCs; the results show that CD44 and S100B were upregulated with no significance, whereas the mRNA levels of other genes were significantly increased (Figure 3E). The positive immunofluorescence staining and upregulated genes related to neural markers both indicated that CLNSCs had the capacity to undergo neural differentiation.

### 2.4. Global Transcriptional Features of CLNSCs

**Figure 4:**
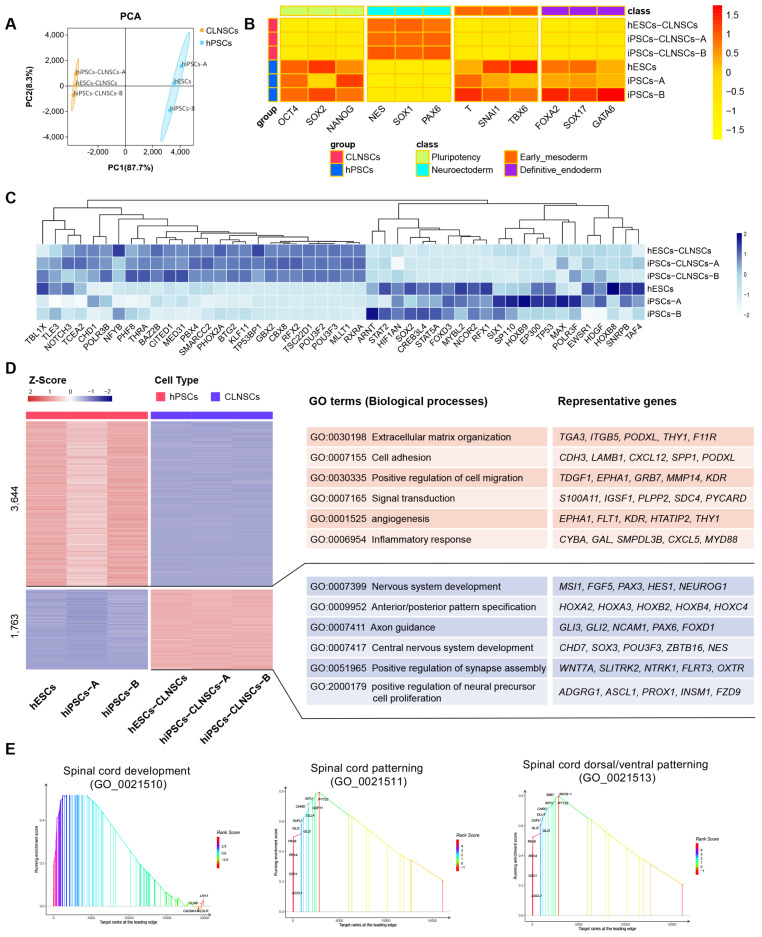
The transcriptome changes in CLNSCs, pre- and post- neural differentiation. (**A**) PCA plot showing hPSCs and CLNSCs. (**B**) Heatmap shows the gene expression levels of pluripotent and three lineage markers in hPSCs and CLNSCs; the scale bar shows Z-score values. (**C**) Heatmap shows the gene expression levels of TFs that are essential for neuroectoderm in CLNSCs and hPSCs; the scale bar shows Z-score values. (**D**) DGEs in CLNSCs compared with hPSCs were represented by heatmap (|log2 (fold change)| > 1, FDR < 0.05), the scale bar shows Z-score values. Significantly enriched biological processes, GO terms, and the representative up-regulated genes in each GO term are listed on the right; the top GO term is enriched in hPSCs and the bottom GO term is enriched in CLNSCs. (**E**) Spinal cord development-associated GO terms in CLNSCs were significantly enriched by GSEA. The peak in the graph divides the curve into two sides. The first side is called the leading edge, and the genes on this side are leading targets that are worth exploring. The abscissa is target ranks, which indicates the ranking of targets, and ordinate is the enrichment score.

To gain insights into the molecular mechanism in CLNSCs, we performed transcriptome analyses by RNA-sequencing (RNA-seq). The violin plot shows the distribution of three hPSC samples, including one hESC cell line and two iPSC cell lines, and three CLNSC samples derived from above three hPSC cell lines, respectively (Figure S4A). Cluster analysis by PCA (principal component analysis) shows that hPSC samples and the CLNSC samples converted from hPSCs were classified into two groups (Figure 4A). We identified 1763 up- and 3645 down-regulated genes in CLNSCs (Figure S4B). The top 40 differential gene expressions (DGEs) are shown in Table S1. Collectively, the transcriptome signatures of CLNSCs showed a significant divergence from undifferentiated hPSCs.

The pluripotency and specific germ layer markers were analyzed and data are shown in a heatmap (Figure 4B). The expression levels of neuroectoderm markers (*NESTIN*, *SOX1*, *PAX6*) were significantly up-regulated, whereas the pluripotency markers (*OCT4*, *NANOG*, but not *SOX2*), early mesoderm markers (*T*, *SNAI1*, *TBX6*), and definitive endoderm markers (*FOXA2*, *SOX17*, *GATA6*) were not expressed or only poorly expressed in CLNSCs.

We examined the expression of neuroectoderm-associated essential transcription factors (TFs) between hPSCs and CLNSCs (Figure 4C). The specific germ layer TFs selected have been described before [49]. The neural development transcription factors such as *POU3F2* and *POU3F3* were significantly up-regulated. *POU3F2* is vital for the self-renewal and maintenance of NSCs while preventing their differentiation [50]. A previous study demonstrated that the activation of *POU3F2* and *POU3F3* was induced by *PAX6* in neurosphere culture [51], and that a high level of *PAX6* expression was also observed in our CLNSC. The expression of *SOX2* existed in both PSCs and NSCs with different gene regulatory networks, while the co-regulation of *SOX2* and *POU3F2* promotes neural development [52].

A Gene Ontology (GO) enrichment analysis was carried out to identify the functions of DEGs; the top 20 GO terms are shown (Table S2). Through the comparative analysis of the results of GO enrichment between hPSCs and CLNSCs, it was found that some development-relevant GO terms, such as anatomical structure morphogenesis, anatomical structure development, system development, multicellular organism development, developmental process, and especially nervous system development (Figure S4C) corresponding to our neural induction process, were enriched in the CLNSCs group. In addition, adhesion-relevant GO terms such as cell adhesion and biological adhesion were also significantly over-represented. We observed the neural identities of CLNSCs in mRNA and protein expression, as described above.

To investigate the global function of upregulated genes in the biological process of hPSCs and CLNSCs, a GO enrichment analysis was performed to describe the biological functions in both groups. The upregulated genes of hPSCs were enriched in terms of extracellular matrix organization, cell adhesion, positive regulation of cell migration, signal transduction, angiogenesis, and inflammatory response, whereas the upregulated genes of CLNSCs were enriched in terms of nervous system development, anterior/posterior pattern specification, axon guidance, central nervous system development, positive regulation of synapse assembly, and positive regulation of neural precursor cell proliferation (Figure 4D).

A Kyoto Encyclopedia of Genes and Genomes (KEGG) pathway enrichment analysis was performed to further evaluate the biochemical metabolic pathway and signal transduction pathway through the interactions of significant enrichment DGEs. The enrichment of the top 20 KEGG pathways is shown (Table S3). DGEs in signaling pathways regulating the pluripotency of stem cells and the Wnt signaling pathway indicated that canonical Wnt signaling was inactivated and *c-MYC* was inhibited, which downregulated the core transcription factors *OCT4* and *NANOG* but maintained the expression of *SOX2*, thus promoting the upregulation of the expression of ectoderm genes *PAX6*, *MESI1*, *HOXB1*, and *NEUROG1* and the transition to neural fate in CLNSCs.

### 2.5. Spinal Cord Region-Specific Gene Expression Pattern of CLNSCs

To further explore the gene function of CLNSCs, the top 10 GO gene sets’ GSEA demonstrating a significant enrichment of gene sets in CLNSCs compared to hPSCs are shown (Table S4). The significant nervous system related spinal cord GO functional terms such as spinal cord patterning (FDR = 0.003903), spinal cord dorsal/ventral pattern (FDR = 0.003506), and spinal cord development (FDR = 0.011614) were enriched by GSEA (Figure 4E).

**Figure 5:**
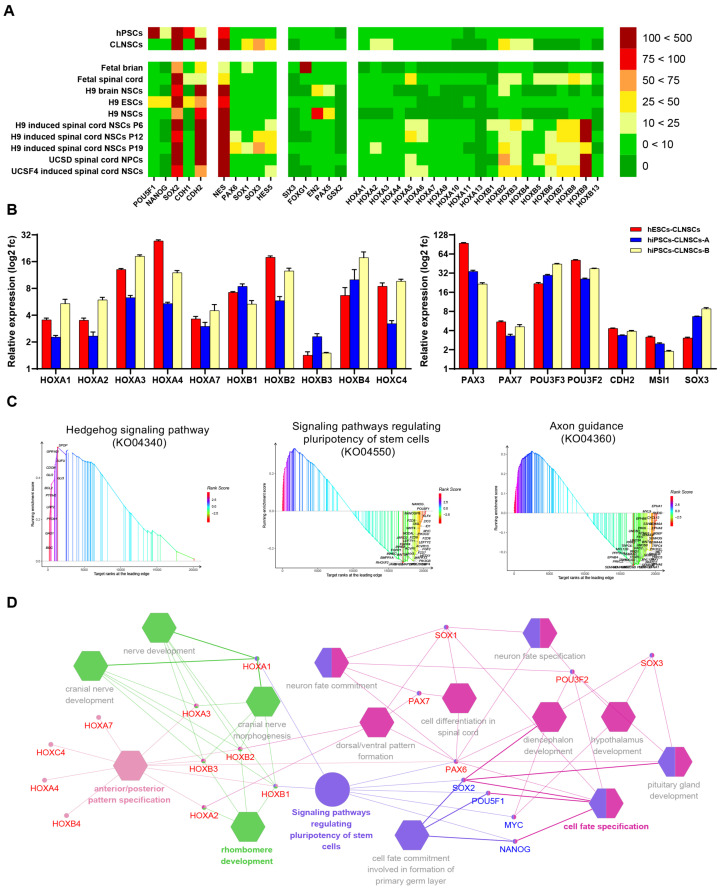
The spinal cord region-specific expression pattern in CLNSCs. (**A**) Expression levels of pluripotent markers, ectoderm markers, brain markers, and *HOX* genes in CLNSCs and fetal brain, fetal spinal, H9 ESCs, H9 NSCs, and induced spinal cord NSCs were compared by RPKM; the scale bar shows RPKM values. (**B**) qRT-PCR analysis of *HOX* genes and the expression of neural genes in different CLNSC lines were presented as log2 (fold change). The relative expression was determined by comparison to the hPSC cell lines. (**C**) KEGG pathway neural development-associated enrichment in NSCs by GSEA. The peak in the graph divides the curve into two sides. The first side is called the leading edge, and the genes on this side are leading targets that are worth exploring. The abscissa is target ranks, which indicates the ranking of targets, and ordinate is the enrichment score. (**D**) The network of candidate targets was constructed using *Cluego* analysis in Cytoscape. The circle indicates the KEGG pathway, and polygons indicate GO terms. Genes labeled red indicate the up-regulated genes and those labeled blue indicate the down-regulated genes.

As the GO terms were highly related to spinal cord development, we focused on the molecular characterization and expression pattern of spinal cord NSCs compared to a previous study. Hierarchical clustering (Figure S4D) and the expression of brain markers and *HOX* genes which control the formation of the brain and spinal cord [53] of CLNSCs were compared with H9 ESCs, fetal brain NSCs, fetal spinal cord NSCs, and induced spinal cord NSCs (Figure 5A), as described before (GSE83107) [34]. According to brain and spinal cord markers, CLNSCs have a different pattern of expression compared with fetal brain and brain specific NSCs but reveal a partially overlapping expression pattern with fetal spinal cord and spinal cord specific NSCs. The *HOX* genes (*HOXA1-4*, *HOXA7*, *HOXB1-4*, *HOXC4*) and neural genes (*PAX3*, *PAX7*, *POU3F2*, *POU3F3*, *CDH2*, *MSI1*, *SOX3*) that were upregulated in CLNSCs were validated by qRT-PCR; the expression patterns of the genes were consistent with those of the RNA-seq analysis (Figure 5B). These results suggest that CLNSCs exhibit a spinal cord region-specific expression pattern.

Core neural TFs were compared between CLNSCs and region-specific NSCs, as described before [34]. The essential TF transcriptome profiles of *POU3F2*, *POU3F3*, *RFX2 TBL1X*, and *ZKSCAN1* in CLNSCs were more similar to the H9-induced spinal cord NSCs, as well as more distinct from the H9 NSCs and brain NSCs (Figure S4E).

### 2.6. Gene Network and Core Pathways in Self-Renewing CLNSCs

For further research on the signaling pathways that are essential for the maintenance of the self-renewal of CLNSCs, we performed a KEGG enrichment analysis using GSEA. The top 10 KEGG gene sets in GSEA demonstrated a significant enrichment of gene sets in CLNSCs compared to hPSCs, as shown in Table S5. The Hedgehog signaling pathway associated with neurodevelopment was the most significantly enriched pathway in KEGG analyzed by GSEA. As reported previously, Hedgehog signaling is crucial for spinal cord development [54]. The signaling pathways regulating the pluripotency of stem cells and axon guidance were also significantly enriched (Figure 5C).

Using the plugins *ClueGO* and *CluePedia*, we integrated candidate targets, including the pluripotent genes *OCT4 (POU5F1)*, *SOX2*, *NANOG*, and *c-MYC*; neural genes *POU3F2*, *SOX1*, *SOX3*, and *PAX6*; and spinal-associated genes *PAX7* and *HOX*, as identified above into networks. The genes were subsequently binned into a pathway, along with the GO terms (Figure 5D). The network revealed that the regulation of the crosstalk between signaling pathways regulating the pluripotency of stem cells and neural-related biological processes might be mediated by *PAX6*, *SOX2*, *HOXA1*, and *HOXB1*, *SOX2*, *POU5F1*, as well as *NANOG* were also binned into the cell fate commitment involved in the formation of the primary germ layer and cell fate specification. Cell differentiation into spinal cords, dorsal/ventral pattern formation, and neuron fate commitment were co-correlated with *PAX6*, *PAX7*, and *SOX1*.

**Figure 6:**
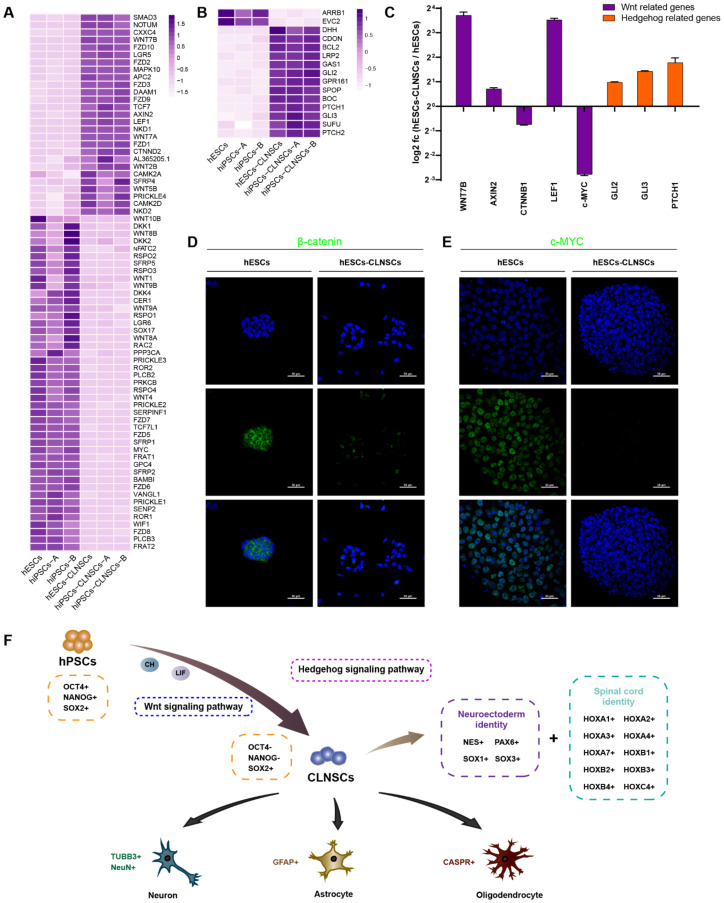
Gene expression patterns in the Wnt and Hedgehog signaling pathways. (**A**) DGEs (|log2 (fold change)| > 1, FDR < 0.05) related to Wnt signaling pathway are shown in the heatmap. (**B**) DGEs (|log2 (fold change)| > 1, FDR < 0.05) related to Hedgehog signaling pathway are shown in the heatmap. (**C**) Relative gene expression of Wnt and Hedgehog signaling pathways in the hESCs-derived CLNSCs compared to hESCs are presented in log2 (fold change). (**D**) Immunofluorescence staining for β-catenin in hESCs (Passage 28) and hESCs-CLNSCs (Passage 18), scale bar: 50 μm. (**E**) Immunofluorescence staining for c-MYC in hESCs (Passage 28) and hESCs-CLNSCs (Passage 18), scale bar: 50 μm. (**F**) Schematic diagram of the conversion process of CLNSCs induced from hPSCs by CHIR-99021 and LIF, and the spinal cord region- specific identities of these NSCs.

Due to supplementing with the Wnt activator CHIR-99021 in the CLNSC culture and Wnt signaling pathway enrichment in DGEs, we observed the expression of the genes that compose the pathway (Figure 6A). The genes involved in the Hedgehog signaling pathway were also analyzed (Figure 6B). We selected several genes, including β-catenin, which is a central player of canonical Wnt/β-catenin, and its target, c-MYC, for validation by qRT-PCR (Figure 6C). The results show that both β-catenin and c-MYC were significantly down-regulated, and AXIN2, a member of the β-catenin destruction complex, was up-regulated. These results suggest that the down-regulation of β-catenin may inhibit the activation of classical Wnt signaling, subsequently down-regulating the expression of its target gene c-MYC and resulting in an exit from the pluripotent state and undergoing cell differentiation. To further verify this conclusion, we performed immunofluorescence staining to observe the location of β-catenin and the changes in the c-MYC protein expression level in hESCs and hESCs-derived CLNSCs. The β-catenin was expressed in nuclear and cytoplasmic fractions in hESCs, but the expression level of β-catenin was significantly decreased in CLNSCs (Figure 6D). The c-MYC expression could also be observed in hESCs but failed in CLNSCs (Figure 6E); the results for the β-catenin and c-MYC protein levels were consistent with those for the mRNA level.

In summary, we established a simple method using the small-molecule inhibitor CHIR-99021 and LIF to generate expanded CLNSCs in vitro. These CLNSCs have spinal cord regional identities with the ability to proliferate and differentiate into the three neural lineages. Through transcriptome analysis, we found that the Wnt signaling pathway and the Hedgehog pathway may play crucial roles in CLNSCs’ self-renewal and proliferation (Figure 6E).

## 3. Discussion

As a part of the central nervous system (CNS), the spinal cord plays a vital role in the transmission of motor and sensory information [55]. Spinal cord injury (SCI) leads to the damage of spinal cord function, resulting in the loss of sensory, motor, and visceral functions [56] and often ending in paralysis [57,58,59,60]. The transplantation of neural stem cells (NSCs) or neural progenitor cells (NPCs) has been suggested as a promising therapeutic strategy to promote the functional recovery of the spinal cord after SCI [61,62]. Moreover, recent studies have demonstrated that tissue-specific NSCs/NPCs have a profound impact on therapeutic efficacy depending on the formation of the phenotypically appropriate interaction between host axons and the neural graft [63,64].

In this study, we described an extremely simple method to generate CLNSCs from hPSCs without multiple stages and the gene modification of neural induction. Expanded NSCs with spinal cord regional identity can be derived by the addition of a small molecule inhibitor CHIR-99021 and a cytokine human LIF to N2B27 medium. During the neural induction process, neural rosettes appeared as early as 2 days, while other methods needed 7 days [44] or 15 days [65], indicating that our neural differentiation strategy has a higher effectiveness in converting hPSCs into CLNSCs. We made use of one hESC cell line and two hiPSC cell lines for this neural induction protocol and obtained similar results, showing that CHIR-99021 and LIF can enable hPSCs exit from pluripotency and contribute to neural differentiation. In our previous study, we found that Activin A, BMP4, CHIR-99021, and LIF, when combined together, can expand the potency of ESCs [66,67], and a recent study also showed that only LIF could maintain the self-renewal of ESCs in mice [68]. Here, we tried to use a single factor to culture CLNSCs and found that CHIR-99021 could maintain the self-renewal of CLNSCs; however, LIF failed (data unshown). These results are consistent with those of a recent study [40]. They indicated that CHIR-99021 plays a critical role in the gastrulation development of hESCs. Other studies have reported that a low dose of CHIR-99021 (0.3 μM [69], 1 μM [70,71]) can maintain the pluripotency of hPSCs, whereas high doses of CHIR-99021 (3 μM [69,70]) promote hPSCs differentiation. Here, 3 μM CHIR-99021 and LIF combined together supported the self-renewal of CLNSCs. The reason for this phenomenon may be that CHIR-99021 is not only a Wnt/β-catenin agonist but also has a complex effect on other targets [72]. Notably, we found the Wnt/β-catenin signaling negatively regulates c-MYC expression in CLNSCs, resulting in neural differentiation and self-renewal. Our findings are consistent with the degradation of β-catenin to maintain NSCs proliferation, as established in a previous study [42]. However, further studies are needed to elucidate mechanisms of β-catenin acting in CLNSCs. Additionally, noncanonical Wnt signaling involves two pathways: the planar cell polarity (PCP) pathway and the Wnt/Ca^2+^ pathway, which also play essential roles in NSCs fate. WNT5A can activate PCP signaling, thereby suppressing Wnt/β-catenin signaling [73]. The up-regulated expression level of WNT5A in CLNSCs is probably also responsible for the degradation of β-catenin. WNT7A deficiency impairs neurogenesis [74,75], indicating that WNT7A is a key element in the regulation of NSCs self-renewal/differentiation [76]; similarly, we observed an up-regulated expression of WNT7A in CLNSCs.

Hedgehog signaling is significant for spinal cord region-specific NSCs; therefore, a previous method for generating spinal cord NSCs was supplemented with Hh1.5 (a potent SHH agonist), CHIR-99021, LDN-193189, SB-431542, FGF2, and FGF8 [34]. In our protocol, Hedgehog signaling is also activated without supplement with SHH agonists. In addition, CLNSCs have no expression of HOX6-9, probably because we did not add the supplement of FGF. According to previous reports, FGF signaling promotes brachial and thoracic spinal features by the activation of HOX6-9 [77,78,79]. FGF signaling regulates cervical spinal identity with downstream HOX genes (HOX6-9) through CDX2 expression, whereas CDX2 expression was not observed in this study; this validated the reasoning described above. Our CLNSCs express HOX genes (HOX1-4) which are similar to induced NSCs by RA (retinoic acid) combined with SAG (agonist of Hedgehog signaling), Wnt3A and bFGF cocktail [80,81]. Previous studies have demonstrated that PAX3 and PAX7 are crucial to aspects of spinal cord development, such as the dorsal part of the developing spinal cord formation, as well as controlling several core cellular processes [82]. The upregulated expression of PAX3/7 in CLNSCs also illustrates that CLNSCs have similar spinal region expression profiles. Interestingly, hierarchical clustering analysis (Figure S4D) indicated that the transcriptome profile of CLNSCs is closer to hPSCs such as hESCs, hiPSCs, and H9 ESCs than to other NSCs.

In summary, we have provided a method that can easily generate CLNSCs with a rostral cervical spinal identity and can be stably passaged over 40 times in vitro. Based on the transcriptome analyses, the corresponding mechanisms in CLNSCs were explored. Our study used three human pluripotent stem cell lines (hESCs and hiPSCs) for the validation of the characteristics and regulatory mechanisms of CLNSCs. These events demonstrate a simple change in the medium support cells transition from pluripotency to neural stem cells. Further investigations are needed to assess the ability of CLNSCs induced by CHIR-99021 and LIF to differentiate into functional spinal interneurons or spinal motor neurons and evaluate the safety of cell transplantation through a tumorigenicity assay.

## 4. Materials and Methods

The human embryonic stem cell line W24 was obtained from Wellcome Trust/Cancer Research UK Gurdon Institute of University of Cambridge. The human induced pluripotent stem cell lines (hiPSCs-A and hiPSCs-B) were preserved in our laboratory after being established from fibroblasts using an approach described before [83]. The study protocols were approved by the Medical Ethics Committee of Inner Mongolia Medical University (YDK202001129, 7 April 2020).

### 4.1. Human Pluripotent Stem Cells Culture

The cells were cultured in mTeSR™1 (StemCell Technologies, Van-couver, BC, Canada) and coated with human vitronectin (Gibco, Gaithersburg, MD, USA). Cells were passaged by 1:10 with 0.02% EDTA (Sigma, Darmstadt, Germany). The 0.2 g EDTA was dissolved in 1 Liter Dulbecco’s phosphate-buffered saline (DPBS, Biological industries, Cromwell, CT, USA).

### 4.2. Neural Induction and Neural Stem Cells Maintenance

Once the hPSCs were cultured at 70–80% confluency in a 24-well plate, the culture medium mTeSR™1 was removed and switched to a neural induction medium. The neural induction medium named CL medium was N2B27 medium supplemented with CHIR-99021 (3 µM, Miltenyi Biotech, Auburn, CA, USA) and human LIF (10 ng/mL, Millipore, Billerica, MA, USA). N2B27 medium: 1:1 mixture of DMEM/F12 medium (Gibco) and neurobasal medium (Gibco), 0.5% N2 (Gibco), 1% B27 (Gibco), 1% Glutamax (Gibco), 1% NEAA (Gibco), 100 µM β-mercaptoethanol (Sigma), and 1% penicillin/streptomycin (Sigma). The CL medium was changed every day before the neural rosette structure was observed [44,46], with neural precursor marker NESTIN and neuroepithelial marker N-cad positive [45] appearing within 2–3 days. Neural rosettes were harvested by centrifuge and dissociated by Accutase (Invitrogen, Carlsbad, CA, USA) to a single-cell suspension, then seeded in a 24-well plate coated with human vitronectin (Gibco). The half volume of CL medium was changed every day for a further 6–7 days and neurospheres [84] were formed. Neurospheres were passaged by the single-cell method described above when they reached an approximately 100–200 μm diameter after 7–8 days. The CLNSCs derived from these hPSCs were maintained with CL medium and stably cultured in vitro over 40 passages.

### 4.3. Differentiation of Neural Stem Cells

A total of 6–7 days after plating, the neurospheres induced from hPSCs (Passage 15) were dissociated by Accutase (Invitrogen) to a single-cell suspension, cells were washed in DPBS and resuspended in Complete NeuroCult™ Differentiation Medium (StemCell Technologies), then vitronectin-coated round glass coverslips in 24-well plates were used to seed cells. The cells were cultured with the NeuroCult™ Differentiation Medium for 10 days to differentiate, and the media were changed every two days. Neurons, astrocytes, and oligodendrocytes differentiated from CLNSCs were detected with immunofluorescence staining.

### 4.4. Alkaline Phosphatase Staining

The cells cultured in 24-well plates were fixed with 4% paraformaldehyde (Solarbio, Beijing, China), and their alkaline phosphatase activity was measured to assess stem cell identity [85]. This was performed by Leukocyte Alkaline Phosphatase Kits (Sigma); the procedure for staining went according to the manufacturer’s protocols.

### 4.5. Karyotype

Cells were treated with 0.2 µg/mL colchicine for 2 hours (h) 15 min before harvest for metaphase chromosomes. Following 2 washes with DPBS, cells were dissociated by Accutase (Invitrogen) and resuspended gently with prewarmed hypotonic solution (0.075 mol/L KCl, Sigma) for 45 min at 37 °C. Then, we pre-fixed cells by adding 1 mL of cold fixative solution (3:1, methanol:glacial acetic acid) to the cell suspension. The cells were centrifuged to discard the supernatant and the cells were fixed 2–3 times using a cold fixative solution. Fixed cells were dropped onto pre-cold clean slides, the slides were dried in an incubator at 70 °C for 2 h, and the chromosome preparations were G-banded using trypsin-EDTA (Biological industries) and stained with Giemsa (Sigma). The slides were observed and photographed using a microscope (Nikon, Tokyo, Japan), and the images were analyzed by LUCIA Cytogenetics (Lucia, Praha, Czech Republic).

### 4.6. Immunocytochemistry

Culture cells were fixed with 4% paraformaldehyde (Solarbio) after being briefly washed three times by DPBS for 20 min. For permeabilized and blocked cells, 1% BSA (Biological industries) and 0.1% Triton X-100 (Sigma) were added to DPBS for 30 min. Cells were incubated with primary antibodies (diluted with the buffer described above) overnight at 4 °C. After washing with DPBS three times (5 min each time), the secondary antibody was added and further incubated at room temperature for 1 h, protected from light. The slides were mounted with a Vectashield mounting medium containing DAPI (Vector Laboratories, Burlingame, CA, USA) after being washed three times by DPBS (5 min each time); finally, the slides were sealed using coverslips with nail polish and imaged using a Nikon confocal microscope. The primary antibodies used were: rabbit polyclonal OCT4 (NOVUS, Littleton, CO, USA, 1:200), rabbit polyclonal NANOG (PeproTech, Princeton, NJ, USA, 1:200), goat polyclonal SOX2 (R&D Systems, Minneapolis, MN, USA, 1:200), rabbit monoclonal NESTIN (Boster, Wuhan, Hubei, China, 1:200), rabbit polyclonal PAX6 (Elabscience, Wuhan, Hubei, China, 1:200), rabbit polyclonal N-Cadherin (Abcam, Cambridge, UK, 1:200), goat polyclonal Brachyury (R&D Systems, 1:100), goat polyclonal SOX17 (R&D Systems, 1:200), mouse monoclonal NeuN (CST, Danvers, MA, USA, 1:100), mouse monoclonal TUBB3 (Bioss, Beijing, China, 1:200), rabbit polyclonal GFAP (Bioss, 1:200), and rabbit polyclonal CASPR (Bioss, 1:200). The secondary antibodies used were Alexa Fluor 488 or Alexa Fluor 568 (Molecular Probes, Eugene, CA, USA).

### 4.7. Real-Time PCR

To extract the total RNA, the RNeasy Plus Mini Kit (QIAGEN, Hilden, Germany) was used following the manufacturer’s instructions. The first-strand cDNA was synthesized using the Reverse Transcription System (Promega, Madison, WI, USA). qRT-PCR was performed using SYBR FAST (Kapa Biosystems, Woburn, MA, USA) according to the instructions on a LightCycler 96 Instrument II (Roche Life Science, Mannheim, Germany). The list of primer sequences is provided in Table S6.

### 4.8. Flow Cytometry

Single-cell suspensions were obtained from different passages of the neurosphere with Accutase (Invitrogen) for 5 min at 37 °C, then the cells were fixed with 4% paraformaldehyde (Solarbio) for 20 min. NESTIN-PerCP-Cy5.5 (BD Biosciences, Mountainview, CA, USA) and CD45-FITC (BD Biosciences) were incubated for 30 min at room temperature, shielded from light. The cell concentration was about 5 × 10^6^ cells per milliliter of DPBS and the antibody dilution was used in accordance with the recommendations of the instructions. Cells were analyzed by the FACS Calibur with CellQuest software (BD Biosciences).

### 4.9. RNA-seq and Analysis

Total RNA was isolated from hPSCs and CLNSCs using the Trizol reagent kit (Invitrogen) according to the manufacturer’s instructions. RNA quality was assessed, enriched mRNA was reverse-transcribed into cDNA with random primers, and second-strand cDNA was synthesized. Then, the cDNA fragments were purified and sequenced using the Illumina HiSeq2500 by Gene Denovo Biotechnology. The FPKM (Fragments Per Kilobase of transcript sequence per Millions) of each gene was calculated to estimate the gene expression levels. The differential expressing genes were compared between the hPSCs control group (hESCs passage 25, hiPSCs-A passage 26, and hiPSCs-B passage 22) and the CLNSCs group (hESCs-derived NSCs passage 25, hiPSCs-A-derived NSCs passage 25, and hiPSCs-B-derived NSCs passage 25) using the DESeq2 software [86]. Genes with the parameters of false discovery rate (FDR) < 0.05 and absolute fold change ≥2 were considered to be differentially expressed. Gene Ontology (GO) term enrichment analysis, KEGG pathway enrichment analysis, and PCA and GSEA analysis were performed using the OmicShare tools, an online platform for data analysis. The batch effects from the RNA-seq between different datasets were removed using the *limma* R package. Clustering analysis was performed using t-SNE analysis with the *Rtsne* R package. Heatmaps in the manuscript were plotted using the *pheatmap* R package (v1.0.12) and *Graphpad prism* (v9.0). Interaction networks of the selecting genes and the identified pathways and GO terms were constructed by the Cytoscape Plugins *ClueGO* and *CluePedia* (v2.5.7).

### 4.10. Statistical Analyses

Statistical analyses were carried out using the GraphPad Prism software (v9.0.0). Data were represented as mean ± SD. Significance differences were measured by unpaired two-tailed Student’s *t* test, and *p* < 0.05 was considered statistically significant.

## Data Availability

The data supporting the findings of this study are available from the corresponding author upon request. RNA-seq data have been submitted to the SRA repository and are available at PRJNA705237.

## Supplementary Materials

The following are available online at https://www.mdpi.com/article/10.3390/ijms22147473/s1.
