# Supplementary material for: Global Transcriptional Analyses of the Wnt-Induced Development of Neural Stem Cells from Human Pluripotent Stem Cells
Source: Int J Mol Sci. 2021 Jul 12;22(14):7473. doi: 10.3390/ijms22147473 (PMC8308016; doi:10.3390/ijms22147473)
Supplement: Supplementary file 1 [file ijms-22-07473-s001.zip › ijms-1250049-supplementary.pdf]

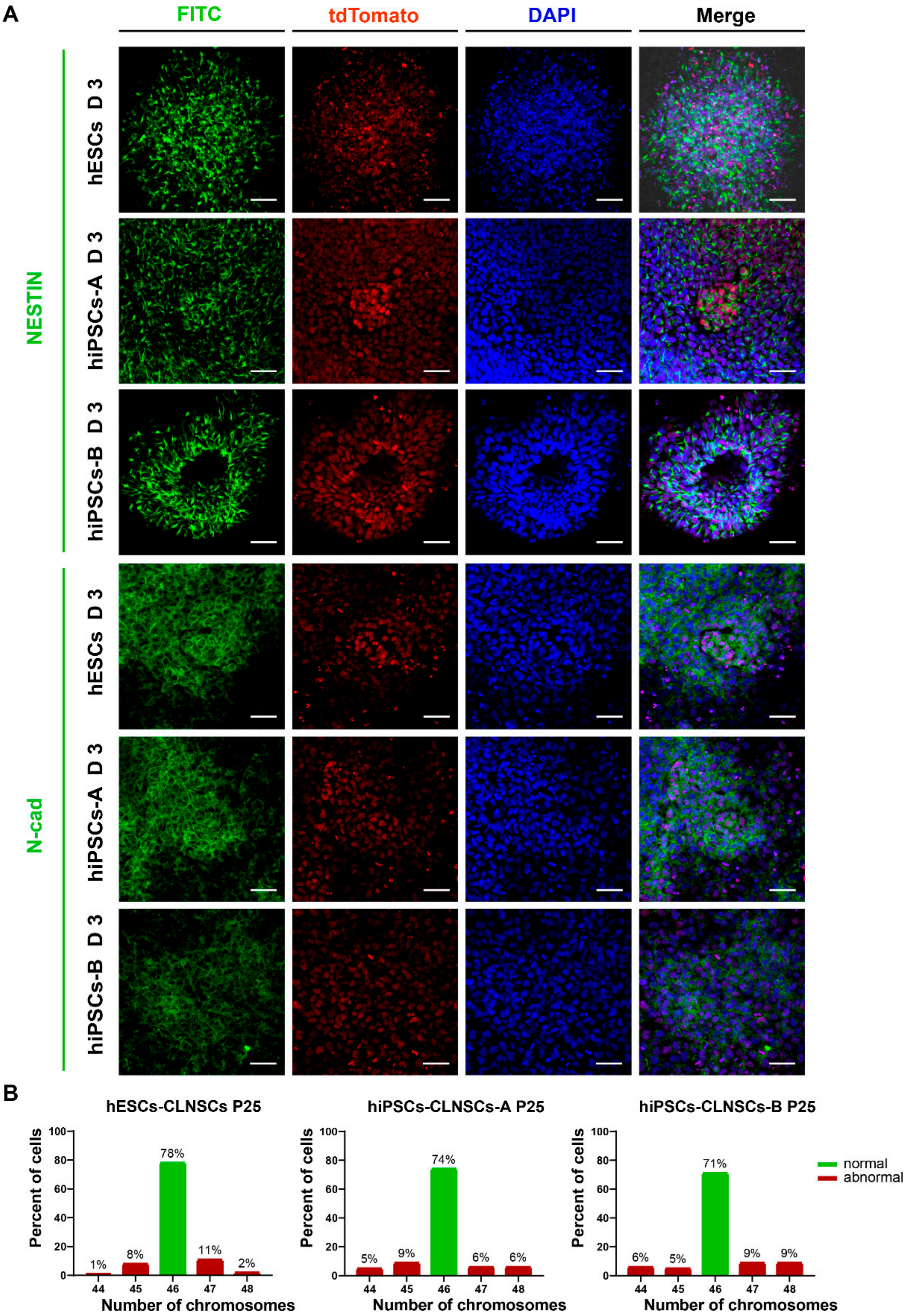
A
FITC
tdTomato
DAPI
Merge
NESTIN
N-cad
hESCs D 3
hiPSCs-A D 3
hiPSCs-B D 3
B
hESCs-CLNSCs P25
hiPSCs-CLNSCs-A P25
hiPSCs-CLNSCs-B P25
Percent of cells
Number of chromosomes
normal
abnormal
1%
8%
78%
11%
2%
5%
9%
74%
6%
6%
6%
5%
71%
9%
9%
44
45
46
47
48

Figure S1: Immunostaining for neural rosettes during CLNSC conversion. (A) Immunofluorescence staining of neural rosette structures for neural marker NESTIN and N-cad in hPSCs on the third day after the treatment for CHIR and LIF, scale bar: 50 μm. (B) Normal and abnormal karyotypes of three CLNSC cell lines (passage 25) were counted, and 100 karyotyping spreads were counted per cell line.

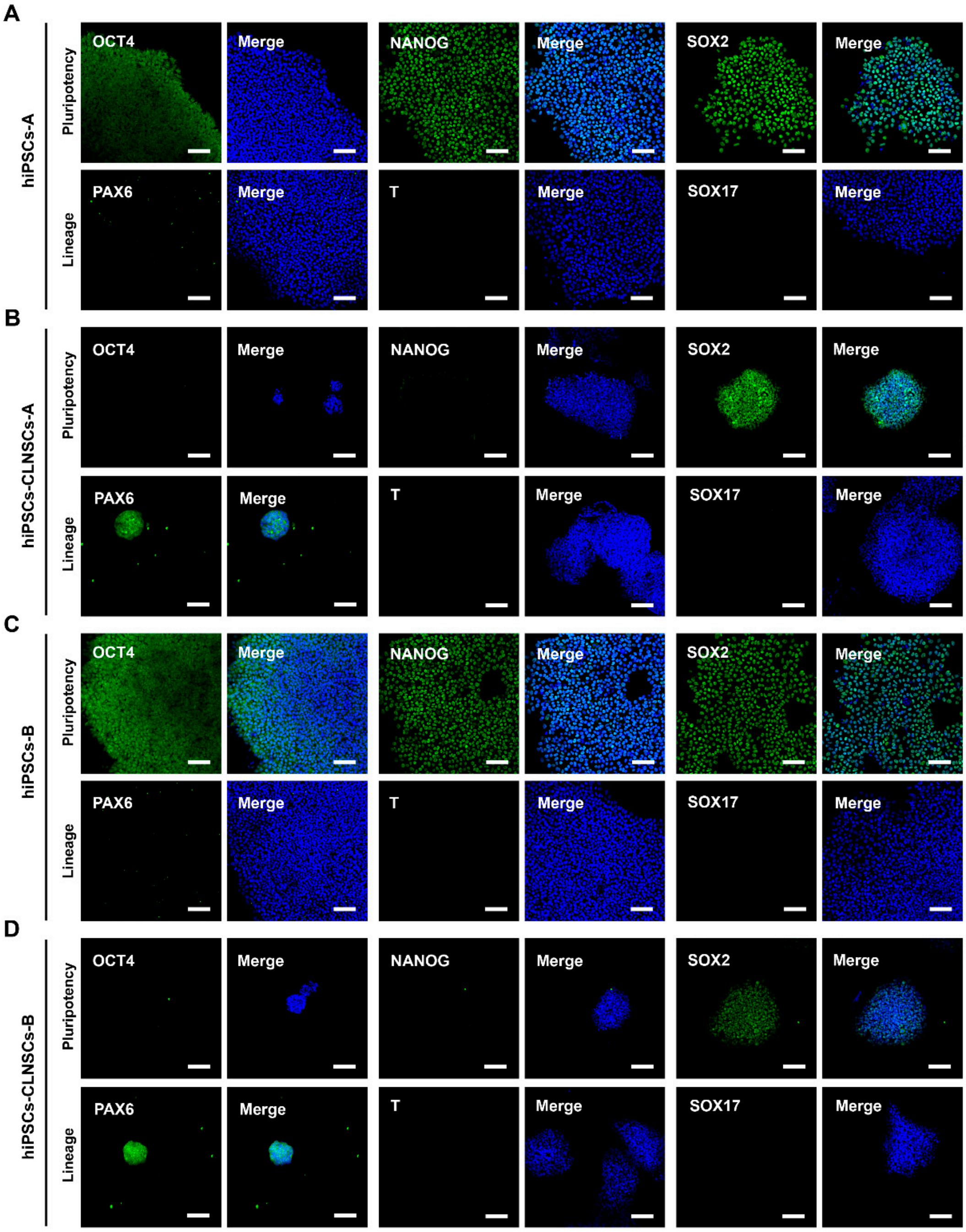

A
hiPSCs-A
Pluripotency
OCT4
Merge
NANOG
Merge
SOX2
Merge
Lineage
PAX6
Merge
T
Merge
SOX17
Merge
B
hiPSCs-CLNSCs-A
Pluripotency
OCT4
Merge
NANOG
Merge
SOX2
Merge
Lineage
PAX6
Merge
T
Merge
SOX17
Merge
C
hiPSCs-B
Pluripotency
OCT4
Merge
NANOG
Merge
SOX2
Merge
Lineage
PAX6
Merge
T
Merge
SOX17
Merge
D
hiPSCs-CLNSCs-B
Pluripotency
OCT4
Merge
NANOG
Merge
SOX2
Merge
Lineage
PAX6
Merge
T
Merge
SOX17
Merge

Figure S2: Immunostaining for pluripotent markers and germ layer markers of iPSCs-A, iPSCs-B and their derived CLNSCs. (A) Immunofluorescence staining for pluripotent markers OCT4, SOX2 and NANOG and lineage markers PAX6, T and SOX17 in hiPSCs-A (passage 26), scale bar: 100 μm. (B) Immunofluorescence staining for pluripotent markers OCT4, SOX2 and NANOG and lineage markers PAX6, T and SOX17 in hiPSCs-CLNSCs-A (passage 25), scale bar: 100 μm. (C) Immunofluorescence staining for pluripotent markers OCT4, SOX2 and NANOG and lineage markers PAX6, T and SOX17 in hiPSCs-B (passage 22), scale bar: 100 μm. (D) Immunofluorescence staining for pluripotent markers OCT4, SOX2 and NANOG and lineage markers PAX6, T and SOX17 in hiPSCs-CLNSCs-B (passage 25), scale bar: 100 μm.

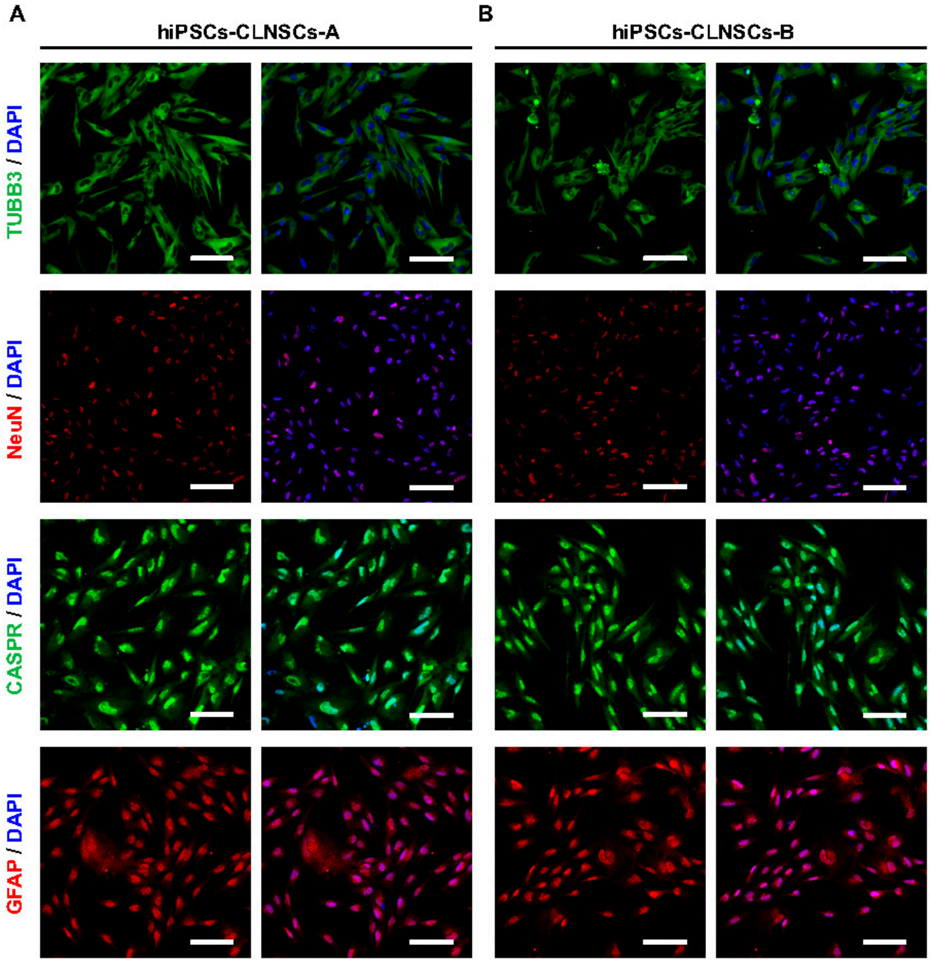

A
B
hiPSCs-CLNSCs-A
hiPSCs-CLNSCs-B
TUBB3 / DAPI
NeuN / DAPI
CASPR / DAPI
GFAP / DAPI

Figure S3: Immunostaining for three neural lineages' differentiation from iPSCs-A- and iPSCs-B-derived CLNSCs.

(A) Immunostaining for neuronal marker TUBB3 (green), neuronal nuclei marker NeuN (red), oligodendrocyte marker CASPR (green) and astrocytic marker GFAP (red) in hiPSCs-CLNSCs-A differentiation, scale bar: 100 μm.

(B) Immunostaining for neuronal marker TUBB3 (green), neuronal nuclei marker NeuN (red), oligodendrocyte marker CASPR (green) and astrocytic marker GFAP (red) in hiPSCs-CLNSCs-B differentiation, scale bar: 100 μm.

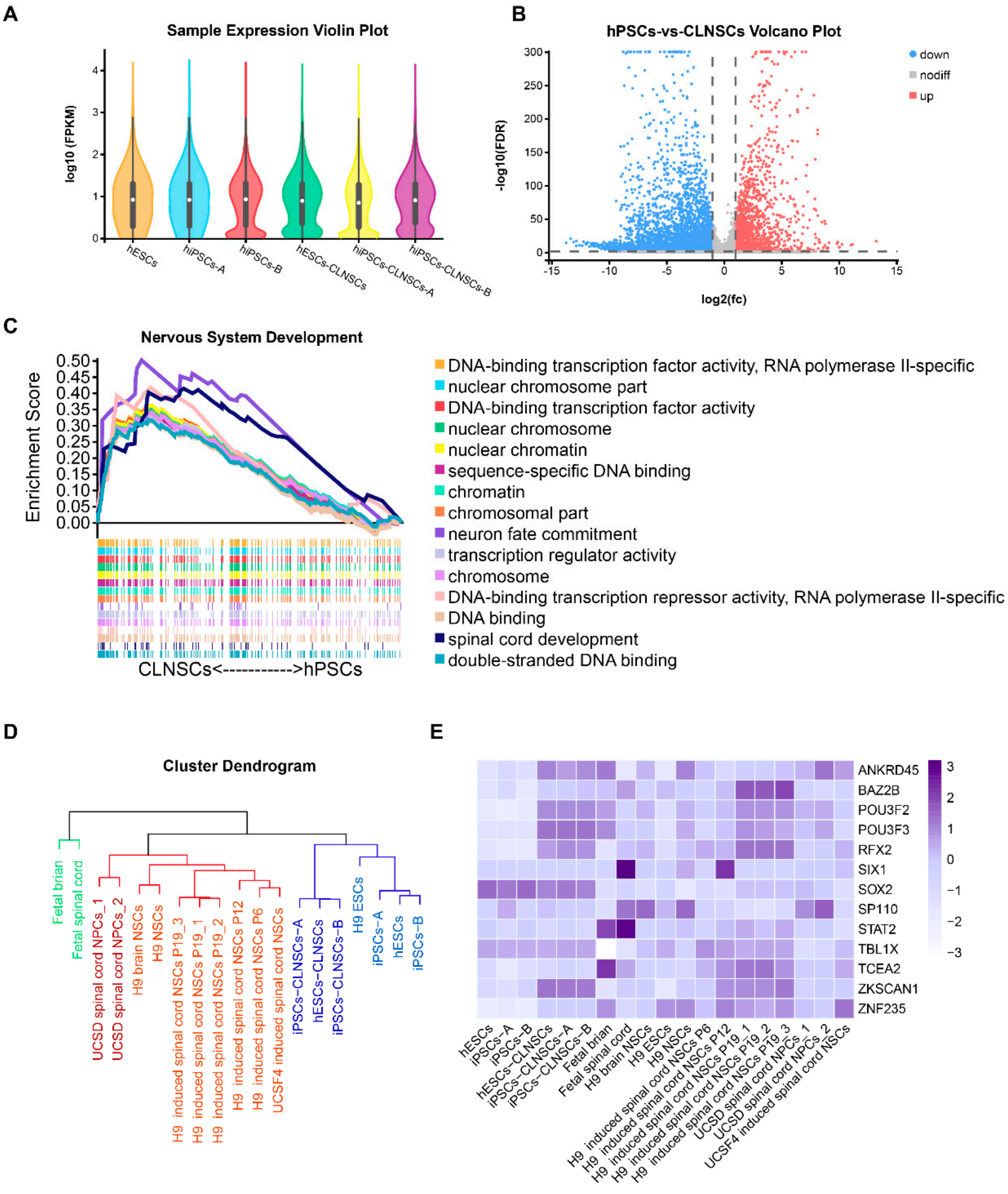
A
Sample Expression Violin Plot
log10 (FPKM)
hESCs
hiPSCs-A
hiPSCs-B
hESCs-CLNSCs
hiPSCs-CLNSCs-A
hiPSCs-CLNSCs-B
B
hPSCs-vs-CLNSCs Volcano Plot
-log10(FDR)
log2(fc)
down
nodiff
up
C
Nervous System Development
Enrichment Score
CLNSCs<----------->hPSCs
DNA-binding transcription factor activity, RNA polymerase II-specific
nuclear chromosome part
DNA-binding transcription factor activity
nuclear chromosome
nuclear chromatin
sequence-specific DNA binding
chromatin
chromosomal part
neuron fate commitment
transcription regulator activity
chromosome
DNA-binding transcription repressor activity, RNA polymerase II-specific
DNA binding
spinal cord development
double-stranded DNA binding
D
Cluster Dendrogram
Fetal brian
Fetal spinal cord
UCSD spinal cord NPCs_1
UCSD spinal cord NPCs_2
H9 brain NSCs
H9 NSCs
H9 induced spinal cord NSCs P19_3
H9 induced spinal cord NSCs P19_1
H9 induced spinal cord NSCs P19_2
H9 induced spinal cord NSCs P12
H9 induced spinal cord NSCs P6
UCSF4 induced spinal cord NSCs
iPSCs−CLNSCs−A
hESCs−CLNSCs
iPSCs−CLNSCs−B
H9 ESCs
iPSCs−A
hESCs
iPSCs−B
E
ANKRD45
BAZ2B
POU3F2
POU3F3
RFX2
SIX1
SOX2
SP110
STAT2
TBL1X
TCEA2
ZKSCAN1
ZNF235
hESCs
iPSCs−A
iPSCs−B
hESCs−CLNSCs
iPSCs−CLNSCs−A
iPSCs−CLNSCs−B
Fetal brian
Fetal spinal cord
H9 brain NSCs
H9 ESCs
H9 NSCs
H9 induced spinal cord NSCs P6
H9 induced spinal cord NSCs P12
H9 induced spinal cord NSCs P19_1
H9 induced spinal cord NSCs P19_2
H9 induced spinal cord NSCs P19_3
UCSD spinal cord NPCs_1
UCSD spinal cord NPCs_2
UCSF4 induced spinal cord NSCs

Figure S4: Transcriptome identity of CLNSCs compared to hPSCs. (A) The violin plot shows the distribution of the abundances per sample. (B) Differential expressed genes in hPSCs compared to CLNSCs were shown by volcano diagram. All differential expression thresholds were set at $| \log 2 \text{ (fold change) } | > 2$ and FDR < 0.05. (C) GO terms in CLNSCs by GSEA using nervous system development gene cluster. The ordinate is the ES value, and the abscissa is the ranks of genes. (D) Dendrogram shows the relationship between CLNSCs, fetal brain, fetal spinal, H9 ESCs, H9 NSCs, and induced spinal cord NSCs. (E) Expression levels of core TFs of neuroectoderm in CLNSCs and fetal brain, fetal spinal, H9 ESCs, H9 NSCs, and induced spinal cord NSCs were compared and displayed by heatmap; the scale bar shows Z-score values.

Table S1. DGEs in CLNSCs compared to hPSCs (top 20 up-regulated and top 20 down-regulated).

Gene	log2(fc)	P Value	Q Value	Description	Regulated trend
VAX2	13.24079	3.92E-18	2.37E-17	ventral anterior homeobox 2	Up
ATP6V1B 1	11.18198	3.60E-13	1.67E-12	ATPase H+ transporting V1 subunit B1	Up
FGF3	11.03388	5.09E-12	2.20E-11	fibroblast growth factor 3	Up
MYBPC1	10.26444	3.27E-13	1.52E-12	myosin binding protein C1	Up
RGR	10.04075	6.26E-09	2.17E-08	retinal G protein coupled receptor	Up
TRIM48	10.00843	9.54E-09	3.27E-08	tripartite motif containing 48	Up
MKRN3	9.903129	8.57E-17	4.90E-16	makorin ring finger protein 3	Up
STMN4	9.827343	2.53E-14	1.26E-13	stathmin 4	Up
LBX1	9.149747	7.34E-16	4.00E-15	ladybird homeobox 1	Up
OR51E2	9.049849	2.40E-08	7.99E-08	olfactory receptor family 51 subfamily E member 2	Up
CD207	9.031586	5.69E-07	1.68E-06	CD207 molecule	Up
UNCX	8.956134	3.58E-07	1.07E-06	UNC homeobox	Up
PRDM13	8.821951	2.15E-44	3.12E-43	PR/SET domain 13	Up
CRYBA1	8.784635	6.97E-12	2.99E-11	crystallin beta A1	Up
HES5	8.757271	1.17E-48	1.87E-47	hes family bHLH transcription factor 5	Up
CXCR5	8.655829	2.98E-08	9.85E-08	C-X-C motif chemokine receptor 5	Up
GSX1	8.50289	2.72E-47	4.21E-46	GS homeobox 1	Up
SCN3A	8.359017	1.90E-16	1.07E-15	sodium voltage-gated channel alpha subunit 3	Up
ASCL1	8.270295	2.20E-16	1.23E-15	achaete-scute family bHLH transcription factor 1	Up
HOXA4	8.256864	1.68E-20	1.13E-19	homeobox A4	Up
AIF1	-13.7317	1.33E-15	7.17E-15	allograft inflammatory factor 1	Down
ACAP1	-13.3299	6.07E-22	4.33E-21	ArfGAP with coiled-coil, ankyrin repeat and PH domains 1	Down
CRB3	-13.0757	8.45E-17	4.83E-16	crumbs cell polarity complex component 3	Down
MT1H	-12.8461	6.83E-11	2.74E-10	metallothionein 1H	Down
DPEP3	-12.8317	2.85E-18	1.73E-17	dipeptidase 3	Down
KLK8	-12.5714	1.01E-11	4.30E-11	kallikrein related peptidase 8	Down
C1orf94	-12.2278	1.59E-17	9.36E-17	chromosome 1 open reading frame 94	Down
SEMG1	-12.0177	3.78E-15	1.98E-14	semenogelin 1	Down
ECSCR	-11.9849	4.08E-13	1.90E-12	endothelial cell surface expressed chemotaxis and apoptosis regulator	Down
MYOZ1	-11.9489	1.19E-13	5.68E-13	myozenin 1	Down
CD177	-11.9006	1.45E-15	7.76E-15	CD177 molecule	Down
C9orf64	-11.8416	1.79E-16	1.01E-15	chromosome 9 open reading frame 64	Down
RBP7	-11.697	7.50E-10	2.79E-09	retinol binding protein 7	Down
OOEP	-11.5035	3.79E-11	1.55E-10	oocyte expressed protein	Down
OVOL2	-11.47	2.70E-13	1.27E-12	ovo like zinc finger 2	Down
CYP4F22	-11.3736	3.85E-15	2.01E-14	cytochrome P450 family 4 subfamily F member 22	Down
TCL1B	-11.3332	3.74E-11	1.53E-10	T cell leukemia/lymphoma 1B	Down
GFY	-11.303	5.22E-12	2.25E-11	golgi associated olfactory signaling regulator	Down
UCMA	-11.2507	6.39E-10	2.39E-09	upper zone of growth plate and cartilage matrix associated	Down
VAV1	-11.13997762	8.01E-15	4.11E-14	vav guanine nucleotide exchange factor 1	Down

[1]The DGEs were sorted by log2 (fold change) from small to large.

Table S2. Top 20 GO term enrichment in CLNSCs compared to hPSCs.

GO ID	Class	Descrption	Q value	Up gene number	Down gene number
GO:0009653	Biological Process	anatomical structure morphogenesis	4.14E-65	408	730
GO:0048856	Biological Process	anatomical structure development	4.14E-65	725	1414
GO:0048731	Biological Process	system development	4.85E-63	645	1178
GO:0007275	Biological Process	multicellular organism development	5.09E-62	692	1286
GO:0032502	Biological Process	developmental process	5.03E-61	760	1485
GO:0032501	Biological Process	multicellular organismal process	9.43E-53	839	1748
GO:0051239	Biological Process	regulation of multicellular organismal process	4.92E-51	409	843
GO:0007399	Biological Process	nervous system development	5.65E-51	437	563
GO:0044459	Cellular Component	plasma membrane part	8.35E-48	299	819
GO:0071944	Cellular Component	cell periphery	8.25E-47	535	1418
GO:0005886	Cellular Component	plasma membrane	6.40E-44	528	1379
GO:0031226	Cellular Component	intrinsic component of plasma membrane	7.86E-43	182	533
GO:0050793	Biological Process	regulation of developmental process	2.18E-42	372	670
GO:0007155	Biological Process	cell adhesion	3.42E-40	166	464
GO:0022610	Biological Process	biological adhesion	4.27E-40	166	466
GO:0005887	Cellular Component	integral component of plasma membrane	1.27E-39	169	510
GO:0048869	Biological Process	cellular developmental process	3.28E-39	551	1034
GO:2000026	Biological Process	regulation of multicellular organismal development	3.74E-39	309	540
GO:0030154	Biological Process	cell differentiation	6.94E-39	532	996

[1]The GO terms were sorted by Q values from small to large (Q values were FDR-adjusted p values).

Table S3. Top 20 KEGG pathway enrichment in CLNSCs compared to hPSCs.

Pathway ID	Pathway term	Q value	DGEs number	Total gene number
ko05200	Pathways in cancer	5.67E-09	218	551
ko04360	Axon guidance	3.83E-08	90	187
ko04550	Signaling pathways regulating pluripotency of stem cells	3.83E-08	76	150
ko04514	Cell adhesion molecules (CAMs)	3.83E-08	77	153
ko05226	Gastric cancer	2.10E-07	75	153
ko04512	ECM-receptor interaction	2.10E-07	51	91
ko04080	Neuroactive ligand-receptor interaction	8.13E-07	142	352
ko05205	Proteoglycans in cancer	1.08E-06	94	212
ko04015	Rap1 signaling pathway	4.06E-06	97	226
ko05412	Arrhythmogenic right ventricular cardiomyopathy (ARVC)	5.80E-06	43	79
ko04510	Focal adhesion	5.80E-06	89	205
ko05033	Nicotine addiction	5.80E-06	27	41
ko04010	MAPK signaling pathway	1.21E-05	121	304
ko05217	Basal cell carcinoma	1.59E-05	36	64
ko05032	Morphine addiction	1.69E-05	48	95
ko04670	Leukocyte transendothelial migration	2.70E-05	56	118
ko04014	Ras signaling pathway	3.03E-05	101	249
ko04024	cAMP signaling pathway	3.56E-05	92	223
ko05224	Breast cancer	3.64E-05	69	156
ko04310	Wnt signaling pathway	6.53E-05	72	167

[1]The KEGG pathway terms were sorted by Q values from small to large (Q values were FDR-adjusted p values).

Table S4. Top 10 GO GSEA in CLNSCs compared to hPSCs.

GO ID	Name	Size	ES	NES	FDR
GO:0042571	immunoglobulin complex, circulating	77	0.636808	2.300412	0
GO:0042073	intraciliary transport	53	0.638603	2.198311	0.005855
GO:0021511	spinal cord patterning	24	0.79223	2.1892	0.003903
GO:0021513	spinal cord dorsal/ventral patterning	22	0.797389	2.172916	0.003506
GO:0048665	neuron fate specification	35	0.685899	2.149398	0.003754
GO:0035735	intraciliary transport involved in cilium assembly	40	0.67574	2.130789	0.00431
GO:0006614	SRP-dependent cotranslational protein targeting to membrane	96	0.571841	2.112587	0.004525
GO:0021510	spinal cord development	113	0.54343	2.063086	0.011614
GO:0048663	neuron fate commitment	70	0.597285	2.06023	0.010712
GO:0031290	retinal ganglion cell axon guidance	21	0.752757	2.037803	0.013288

[1]The GO terms were sorted by Q values from small to large (Q values were FDR-adjusted p values).

Table S5. Top 10 KEGG GSEA in CLNSCs compared to hPSCs.

Pathway ID	Name	Size	ES	NES	FDR
KO04340	Hedgehog signaling pathway	51	0.547081	1.818063	0.034158
KO00100	Steroid biosynthesis	23	0.552454	1.503139	0.306284
KO05217	Basal cell carcinoma	64	0.4328	1.495431	0.216867
KO03460	Fanconi anemia pathway	54	0.412743	1.396622	0.342514
KO01522	Endocrine resistance	104	0.35261	1.342688	0.406275
KO04550	Signaling pathways regulating pluripotency of stem cells	150	0.334518	1.331707	0.366338
KO03010	Ribosome	143	0.330966	1.306256	0.373543
KO05224	Breast cancer	156	0.338367	1.299358	0.34358
KO04360	Axon guidance	187	0.315694	1.272924	0.364486
KO05226	Gastric cancer	153	0.319472	1.26716	0.341285

[1]The KEGG terms were sorted by Q values from small to large (Q values were FDR-adjusted p values).

Table S6. Forward and reverse primers for qRT-PCR.

Gene ID	Symbol	Primers	Sequences (5′-3′)
2597	GAPDH	Forward	CTCTGCTCCTCCTGTTCGAC
		Reverse	TTAAAAGCAGCCCTGGTGAC
5460	OCT4	Forward	ATGCATTCAAACTGAGGTGCCTGC
		Reverse	CCCTTTGTGTTCCCAATTCCTTCC
6657	SOX2	Forward	GCTGCAAAAGAGAACACCAATCCC
		Reverse	AAACTTCCTGCAAAGCTCCTACCG
77923	NANOG	Forward	GGTAGAAATTGGGGTTTAGAAAT
		Reverse	TACAAAAAACAAACAACTTCCC
10763	NES	Forward	GCACCTCAAGATGTCCCTCAG
		Reverse	CTGGGAGCAAAGATCCAAGAC
5080	PAX6	Forward	GTACTGAATGACTCAACTGCTCGG
		Reverse	CTTTAGAAGGAAGCGACACTCTGC
6656	SOX1	Forward	CAACCAGGACCGGGTCAAACG
		Reverse	GCCTCGGACATGACCTTCCACT
3198	HOXA1	Forward	CTCAAGTTGTGGTCCAAGCTAT
		Reverse	TGGGTCTGCTTCCTGATTTAAC
3199	HOXA2	Forward	ACTCCTTTGACCAGGTGGTTTTGC
		Reverse	ACTTTCTTGCAGGCCTCATACTGC
3200	HOXA3	Forward	AAAAAGCGACCTACTACGACAG
		Reverse	GCTGATTGGCATTATAAGCGAA
3201	HOXA4	Forward	TCCACTTCAATCGATACCTGAC
		Reverse	TCTTTCTTCCACTTCATCCTCC
3204	HOXA7	Forward	ACTACCTATTTTGTGCTGGCTGGC
		Reverse	GAGAAGGAGGGATTGATTCTAGGG
3211	HOXB1	Forward	GGTCAAGATTTGGTTCCAGAACCG
		Reverse	ATTGGTGGCTAGGTTCAGTTCAGG
3212	HOXB2	Forward	GATGAAAGAGAAGAAATCCGCC
		Reverse	AAGTGGAATTCCTTCTCCAGTT
3213	HOXB3	Forward	GAAAGCCACCTACTACGACAA
		Reverse	TCGAAGCCGAAGCCATTG
3214	HOXB4	Forward	AAAAAGAGAGACTCAGAGACCCGG
		Reverse	CTGGGAGGGGCACATTTTATTTCC
3221	HOXC4	Forward	GGGTGAATTTCAGGGGAAATGAGG
		Reverse	CTCAAACTGAACAGCTCTGAGAGG
5077	PAX3	Forward	GGTGTTTTTATCAACGGCAGG
		Reverse	CAATTTTCTTCTCCACGTCAGG
5081	PAX7	Forward	CTGCGTCTCCAAGATTCTTTG
		Reverse	GTTTTCCCTCTTGTACTCCTCA
5454	POU3F2	Forward	CTGGAGAGCCATTTCCTCAAAT
		Reverse	TCTGTCTCCTGTTACAAAACCA
5455	POU3F3	Forward	GGTGGACGATGCCTAAAGA
		Reverse	GCAACGGATTGAGGAGAGA
1000	CDH2	Forward	CGATAAGGATCAACCCCATACA
		Reverse	TTCAAAGTCGATTGGTTTGACC
4440	MSI1	Forward	GGGACTCAGTTGGCAGACTAC
		Reverse	CTGGTCCATGAAAGTGACGAA
6658	SOX3	Forward	GATAAGCCTACCCTTCCCGC
		Reverse	GTGTCCCTACGGGGTTCTTG
7477	WNT7B	Forward	GCAAGTGGATTTTCTACGTGTT
		Reverse	ATCTTGTTGCAGATGATGTTGG
8313	AXIN2	Forward	CTCCGAGCTCACACTCAATTC

		Reverse	GACAGGTGATCGTCCAGTATC
1499	CTNNB1	Forward	TGGATTGATTCGAAATCTTGCC
		Reverse	GAACAAGCAACTGAACTAGTCG
51176	LEF1	Forward	TCCCTCATCCAGCTATTGTAAC
		Reverse	CAAGAATCTGGTTGATAGCTGC
4609	c-MYC	Forward	CGACGAGACCTTCATCAAAAAC
		Reverse	CTTCTCTGAGACGAGCTTGG
2736	GLI2	Forward	CAAGAAGCCAAAAGTGGGATC
		Reverse	CAGAATGAGGCTCGTAATGGTA
2737	GLI3	Forward	CAGTATGGGAACTGTCTCAACA
		Reverse	CTTCAGCTTTGAGGCTTGAATC
5727	PTCH1	Forward	TTTTCTGCTGTTTTACAAGCCC
		Reverse	CATGGTAATCTGCGTTTCATGG
10381	TUBB3	Forward	GCCTGACAATTTCATCTTTGGTC
		Reverse	CAGTCGCAGTTTTCACACTCCTT
146713	NeuN	Forward	CCGAGTGATGACCAACAAGAAG
		Reverse	CGCAGCCCGAAATGTATTATAC
1103	ChAT	Forward	GTCAAAATGGCTTCCAACGAG
		Reverse	GTCCAGGCATACAAGGCAGAT
3110	HB9	Forward	TCAAGCTCAACAAGTACCTGTCG
		Reverse	TTGCTGCGTTTCCATTTCATC
338917	CHX10	Forward	GATCGAAAAATGTCCAAATCTGC
		Reverse	TTCGTTGAATGCCTTCTCCAG
5076	PAX2	Forward	ACAAGATTGCTGAATACAAACGAC
		Reverse	GGTGGAAAGGCTGCTGAACT
2670	GFAP	Forward	CACGCAGTATGAGGCAATGGC
		Reverse	GGTAGTCGTTGGCTTCGTGCT
960	CD44	Forward	CCCATCCCAGACGAAGACAGT
		Reverse	ACCAGCCATTTGTGTTGTTGTGT
6285	S-100B	Forward	CTGAAGAAATCCGAACTGAAGG
		Reverse	CTGGAAGTCACATTCGCCGT
1464	NG2	Forward	CGTATTCTCAGTGAAGAGCCCA
		Reverse	ACATCCAGCGAGAAGGCATC
8506	CASPR	Forward	AGATAGACTTAATGAAGAAGCACCG
		Reverse	TTGTGCCCTCGCTGGTAGAAC
1267	CNP	Forward	TCTACTTCGGCTGGTTCCTGAC
		Reverse	TGACCAAGTCCATCTTCTCCCT
